# One-pot hydrothermal oxidation enables in situ construction of CDs/Ni(OH)_2_ composite for electrocatalytic oxygen evolution

**DOI:** 10.3389/fchem.2025.1656451

**Published:** 2025-09-04

**Authors:** Hui Wang, Weijuan Xu, Xuan Han, Yue Yan, Bingxian Zhu, Zhiyuan Wang, Libo Wang, Qingshan Zhao, Mingbo Wu

**Affiliations:** 1 State Key Laboratory of Heavy Oil Processing, College of Chemistry and Chemical Engineering, China University of Petroleum (East China), Qingdao, China; 2 College of Chemical Engineering, Qingdao University of Science and Technology, Qingdao, China

**Keywords:** nickel hydroxide, carbon dots, in situ growth, oxygen evolution reaction, electrocatalysis

## Abstract

Electrochemical water splitting is a promising solution to energy challenges, yet the kinetically sluggish oxygen evolution reaction (OER) at the anode demands highly active and cost-effective catalysts. Herein, we develop a facile one-pot hydrothermal oxidation strategy to simultaneously achieve the oxidative cleavage of petroleum coke into nanoscale carbon dots (HO-CDs) and the *in situ* growth of nickel hydroxide (Ni(OH)_2_) on nickel foam (NF), yielding an HO-CDs-Ni(OH)_2_/NF composite catalyst. The in situ-formed HO-CDs efficiently modulate Ni(OH)_2_ crystallization by suppressing oriented growth to create a nanostructure with abundant active sites. This synergistic interplay significantly enhances both active site accessibility and charge transfer efficiency, leading to exceptional OER performance. The optimized HO-CDs-Ni(OH)_2_/NF catalyst delivers an overpotential of 353 mV at a current density of 50 mA cm^-2^ with a small Tafel slope of 81.2 mV dec^−1^. Furthermore, it demonstrates excellent stability, retaining 92% of its initial current density after a 24-h chronoamperometric test. This work presents a straightforward approach for designing high-performance transition metal-based electrocatalysts through carbon dot-mediated crystal engineering via *in situ* incorporation.

## 1 Introduction

The escalating global energy demand, driven by rapid population growth and economic development, has intensified pressure on finite fossil fuel resources while exacerbating greenhouse gas emissions and environmental degradation (Feng et al., 2023; Yu et al., 2021). These challenges necessitate urgent advances in converting and storing renewable energy efficiently. Among these, electrochemical water splitting coupled with the OER and rechargeable metal-air batteries represent promising avenues due to their operational simplicity and efficiency (Liu et al., 2022; Meng et al., 2024; Zhang W. et al., 2024). However, the OER inherently requires a multi-electron transfer and O–O bond formation, which imparts sluggish kinetics, ultimately governing the overall efficiency of the energy conversion system (Chen et al., 2023; Wu et al., 2024; Zhang R. et al., 2023). While a wide range of materials have been explored for the OER, catalysts based on precious metals, specifically ruthenium and iridium oxides (RuO_2_, IrO_2_), remain the most effective benchmark catalysts, whereas their scarcity and prohibitive cost hinder large-scale implementation (Gao et al., 2024; Tan et al., 2022). Thus, developing cost-effective, earth-abundant alternatives with high catalytic activity is paramount for sustainable hydrogen production.

Numerous current studies are devoted to developing OER electrocatalysts based on transition metal alloys (Putri et al., 2023), oxides (Ma W. et al., 2024), and hydroxides (Chu et al., 2024; Shi et al., 2023) as replacements for RuO_2_ and IrO_2_. Transition metals and their compounds have been identified as a promising class of electrocatalytic materials, offering advantages such as tunable morphologies, facile synthesis, and competitive catalytic performance (Wang et al., 2023; Zhang L. et al., 2024; Zuo et al., 2024). Particularly, Ni(OH)_2_ has demonstrated considerable potential as an efficient OER electrocatalyst. It is recognized as one of the most effective OER catalysts in alkaline electrolytes, and as such has attracted considerable attention in recent years (Yao et al., 2024; Zhang C. et al., 2024). For instance, Lv et al. constructed WN-Ni(OH)_2_ nanowires via hydrothermal growth, nitridation, and electrodeposition, which exhibited an overpotential of 339 mV for OER at a current density of 100 mA cm^-2^ (Lv et al., 2020). Mei et al. fabricated 2D BP/Ni(OH)_2_ heterostructures (BNHNSs) via coupling wet-chemically synthesized Ni(OH)_2_ nanosheets with liquid-exfoliated black phosphorus nanosheets, exhibiting an overpotential of ultralow 297 mV for OER at a current density of 10 mA cm^-2^ (Mei et al., 2022). Despite these advances, the widespread application of Ni(OH)_2_ remains constrained by inherent limitations, including the insufficient electrical conductivity and a scarcity of highly active sites.

Carbon dots (CDs), a burgeoning class of zero-dimensional nanomaterials, offer unique potential to address these challenges. Their rapid charge transfer kinetics, abundant surface functional groups, and large specific surface area enable dual functionality as conductive additives and morphological modulators (Cao et al., 2022; Hong et al., 2023; Ma et al., 2024b; Liu Y. et al., 2021; Wu L. et al., 2024). Tang et al. engineered a CDs-incorporated NiFe-MOF electrocatalyst (NiFe-BDC/CDs) via hydrothermal synthesis. The intercalation of CDs strengthened O 2p-metal 3days hybridization to activate the lattice oxygen for OER, achieving an ultralow overpotential of 250 mV at a current density of 100 mA cm^-2^ (Tang et al., 2025). Integrating Ni(OH)_2_ with CDs could leverage strong interactions within the multi-component nanocomposite to further promote intermolecular electron transfer, which is conducive to enhancing the OER performance of Ni(OH)_2_. Despite the demonstrated potential of CDs in enhancing the OER performance of electrocatalysts, the integration of CDs with Ni(OH)_2_ to boost its OER activity remains unexplored. Moreover, conventional stepwise synthesis, which involves preparing CDs before compositing, not only introduces multi-step complexity but also weakens interfacial bonding, inducing particle aggregation that severely restricts active site utilization. In contrast, *in situ* strategies that synchronize CDs formation with catalyst growth can streamline fabrication and strengthen interfacial electron coupling at the atomic level, mitigating aggregation and mass transfer limitations to unlock higher intrinsic activity (Kashinath and Byrappa, 2022; Teng et al., 2020).

Herein, through a novel one-pot hydrothermal oxidation strategy using petroleum coke as the carbon source, NF as dual-functional substrate/Ni source, and H_2_O_2_ as the oxidant, we concurrently achieve both oxidative cleavage of petroleum coke into hydroxyl-functionalized carbon dots (HO-CDs) and *in situ* growth of Ni(OH)_2_ on NF. This process directly yields an integrated HO-CDs-Ni(OH)_2_/NF electrocatalyst, wherein in situ-formed HO-CDs critically modulate Ni(OH)_2_ crystallization, suppressing oriented growth to foster a nanostructure rich in active sites. Owing to the synergistic interplay between enhanced active site exposure and promoted charge transfer efficiency, the optimized HO-CDs-Ni(OH)_2_/NF catalyst achieves an ultralow overpotential of 353 mV at 50 mA cm^-2^ with a small Tafel slope of 81.2 mV dec^−1^ while retaining 92% activity after 24 h of operation, establishing a novel approach for high-performance electrocatalyst design via *in situ* CDs incorporation-mediated crystal growth modulation.

## 2 Materials and methods

### 2.1 Chemicals and reagents

Petroleum coke was sourced from China National Petroleum Corporation. H_2_O_2_ (30.0 wt%), acetone (99.5 wt%), hydrochloric acid (36.0–38.0 wt%), KOH (95 wt%), and anhydrous ethanol (Analytical Reagent grade) were procured from Sinopharm Chemical Reagent Co., Ltd. NF was commercially acquired from Kunshan Maipengchen Electronics Technology Co., Ltd.

### 2.2 Synthesis of the catalysts

Synthesis of HO-CDs. Petroleum coke powder (1.0 g) was mixed with 10 mL 30 wt% H_2_O_2_ and 30 mL deionized water in a beaker. After 30 min of magnetic stirring at room temperature, the homogeneous suspension underwent hydrothermal treatment at 140 °C for 12 h in a 100 mL Teflon-lined autoclave. After ambient cooling, the resulting black solution was filtered under vacuum to remove unreacted particles. The filtrate underwent dialysis (MWCO: 3,000 Da) against deionized water for 72 h, and the final product was obtained as HO-CDs powder via 48 h freeze-drying.

Synthesis of HO-CDs-Ni(OH)_2_/NF and Ni(OH)_2_/NF. NF pieces (1 cm × 4 cm) were ultrasonicated sequentially in acetone and 3 M HCl (30 min each) to remove impurities, followed by deionized water and ethanol (15 min each), then vacuum-dried at 60 °C before use. The pretreated NF was introduced into the autoclave during the HO-CDs hydrothermal synthesis to yield the HO-CDs-Ni(OH)_2_/NF catalyst. Ni(OH)_2_/NF was obtained using the same procedure without the addition of petroleum coke.

Synthesis of HO-CDs + Ni(OH)_2_/NF. 100 mg of as-prepared HO-CDs were dispersed in a mixed solution containing 10 mL of 30 wt% H_2_O_2_ and deionized water (30 mL), forming a suspension under stirring. The suspension was transferred into an autoclave, into which the pretreated NF was immersed. Hydrothermal treatment was performed at 140 °C for 12 h. After cooling to room temperature naturally, the resulting product was collected and sequentially washed with ethanol and deionized water three times to remove residual impurities, followed by vacuum drying at 60 °C for 12 h to obtain the final HO-CDs + Ni(OH)_2_/NF catalyst.

### 2.3 Characterizations

The morphology of the prepared catalysts was examined using scanning electron microscopy (SEM, Hitachi S4800, Japan) and transmission electron microscopy (TEM, JEOL JEM-2100UHR, Japan). Atomic force microscopy (AFM, Multimode8 SPM, Bruker, United States) characterized the morphology and particle size of CDs. Particle size distribution was determined using nanoparticle size analysis (SZ-100V2, Horiba). Phase analysis was performed using X-ray diffraction (XRD, X’Pert PRO MPD, PANalytical, Netherlands); surface composition and chemical states were investigated by X-ray photoelectron spectroscopy (XPS, PHI 5000 VersaProbe, PerkinElmer, United States), using the C 1s peak at 284.8 eV as reference. Raman spectroscopy was performed on a Renishaw inVia 2000 spectrometer (Renishaw inVia 2000, UK). Fourier transform infrared (FTIR) spectroscopy (NEXUS, Thermo Scientific, United States) was performed to analyze molecular structures.

### 2.4 Electrocatalytic OER measurement

Electrochemical measurement was conducted on a CHI760E workstation (CH Instruments, China) using a standard three-electrode configuration. The working electrode was functioned as by the catalysts (1 cm × 1 cm), with a graphite rod counter electrode and an Ag/AgCl reference electrode being employed. 1.0 M KOH electrolyte was utilized in the tests. Linear sweep voltammetry (LSV) was performed at 5 mV s^-1^ in O_2_-saturated electrolyte. Electrochemical double-layer capacitance (C_dl_) was determined from cyclic voltammetry (CV) conducted in the non-faradaic region at scan rates of 20–120 mV s^-1^. A linear slope equating to C_dl_ was yielded by half the difference between anodic and cathodic current densities (1/2Δj) *versus* scan rate. Cycling stability was evaluated through 3000 CV cycles carried out at 100 mV s^-1^. Alternatively, current retention over 24 h was measured by chronoamperometry conducted at a fixed potential to assess long-term stability.

## 3 Results and discussion

**FIGURE 1:**
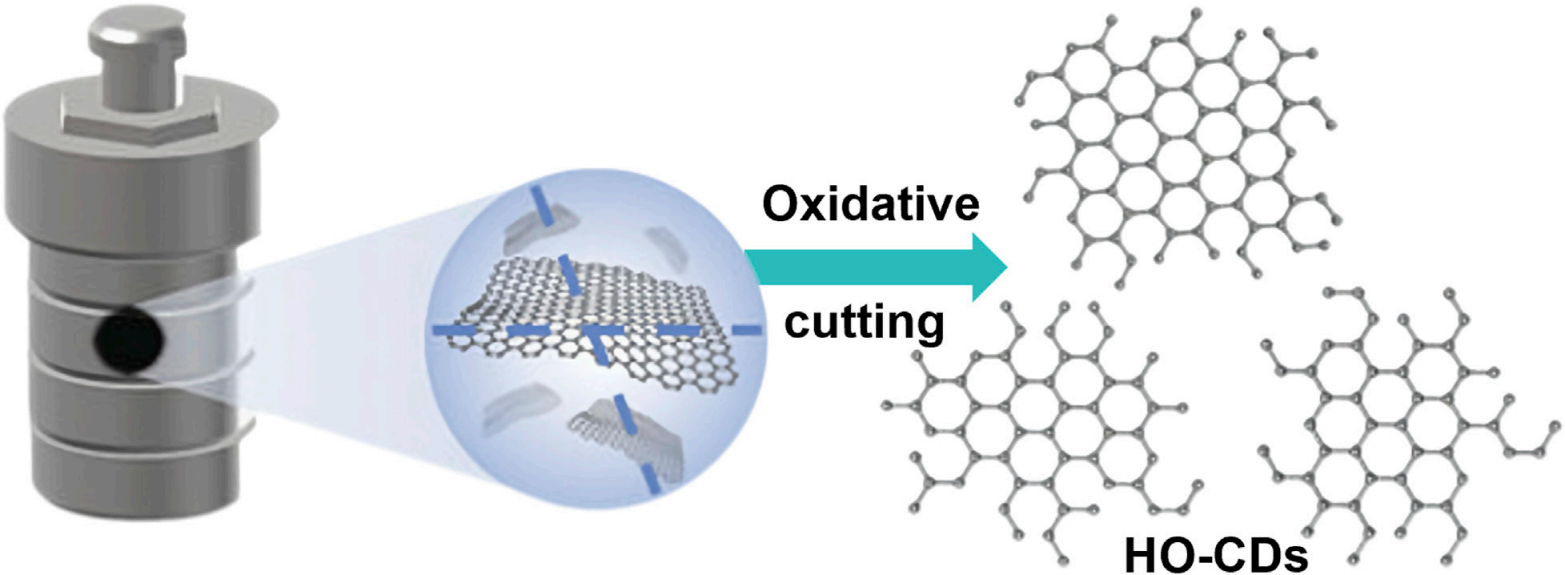
Schematic illustration of the synthesis mechanism of HO-CDs.

Figure 1 delineates the synthesis mechanism of HO-CDs from petroleum coke via H_2_O_2_-mediated hydrothermal oxidation. Petroleum coke powder dispersed in H_2_O_2_ solution undergoes oxidative cleavage under hydrothermal conditions. During this process, H_2_O_2_ decomposition generates highly reactive hydroxyl radicals (∙OH), which preferentially attack thermodynamically unstable organic amorphous carbon chains due to their lower bond dissociation energies compared to crystalline carbon domains. This selective oxidation converts amorphous carbon into gaseous products (CO_2_/H_2_O), while preserving graphitic structures that evolve into HO-CDs with uniform size distribution. Critically, H_2_O_2_ serves as a bifunctional oxidant in this synchronous process: it decomposes to generate ∙OH radicals responsible for cleaving petroleum coke into HO-CDs, while simultaneously oxidizing the NF substrate to release Ni^2+^ ions essential for the nucleation and growth of Ni(OH)_2_.

**FIGURE 2:**
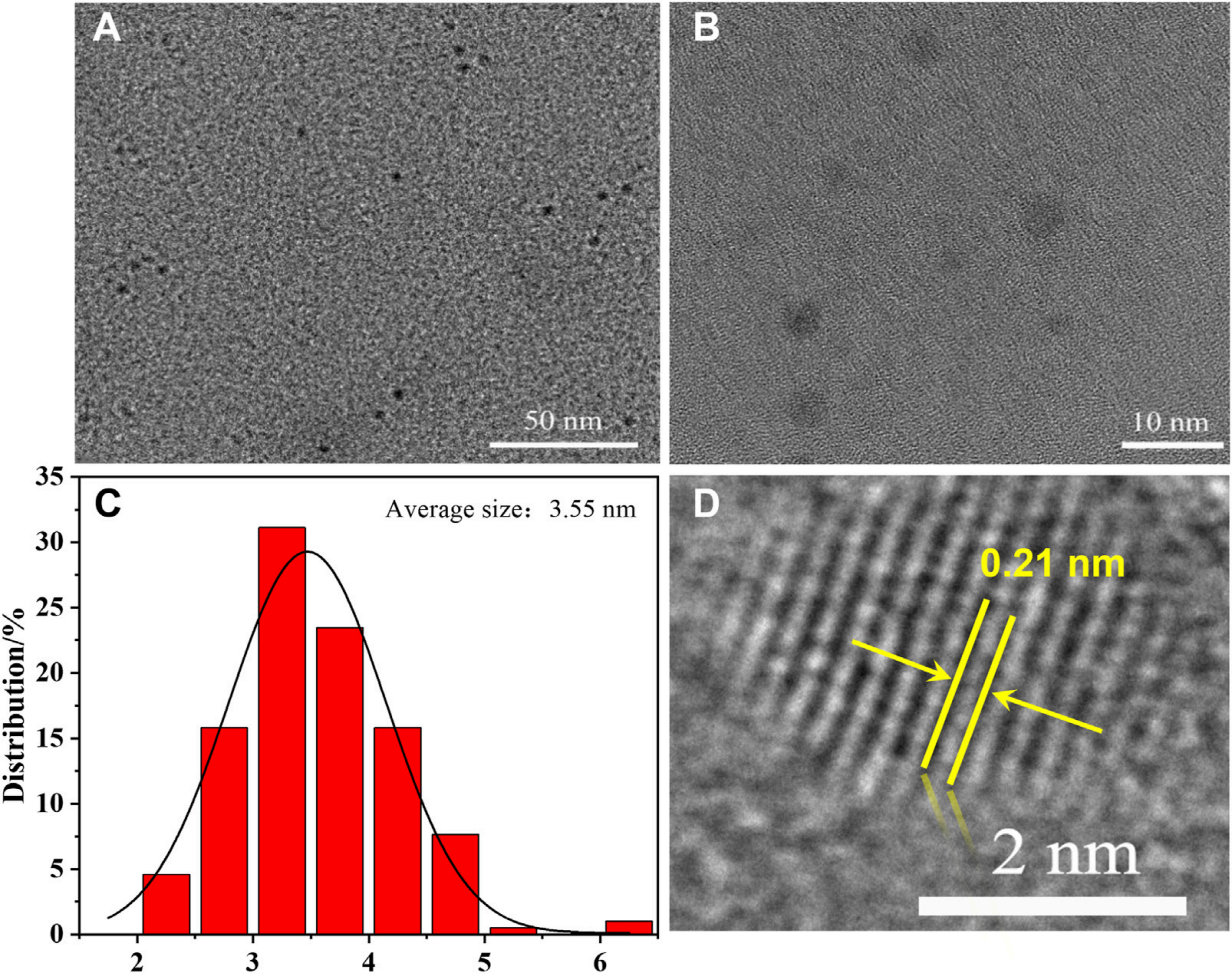
**(A,B)** TEM images and **(C)** corresponding particle size distribution diagram, **(D)** HRTEM image of HO-CDs.

Figures 2A,B display the TEM images of HO-CDs, revealing quasi-spherical morphologies with relatively uniform particle dispersion and no significant aggregation observed. Statistical analysis of the particle size distribution (Figure 2C) further indicates an average particle size of 3.55 ± 0.06 nm with a relatively narrow size distribution. To further investigate the crystal structure of HO-CDs, high-resolution transmission electron microscopy (HR-TEM) analysis was performed on the sample, as shown in Figure 2D. Distinct lattice fringes can be observed, demonstrating a well-defined crystalline structure. The measured lattice spacing of 0.21 nm corresponds to the (100) plane of graphite, confirming the graphitic structural characteristics of HO-CDs and verifying their successful synthesis. Simultaneously, the XRD and Raman spectra (Supplementary Figure S1) of HO-CDs compared to the raw petroleum coke indicate an oxidative cutting process occurred during preparation, leading to increased crystalline disorder. Nevertheless, the graphitic phase structure remains preserved. Furthermore, AFM characterization (Supplementary Figure S2) further corroborates these findings, showing consistency with the TEM results. Collectively, the TEM, HRTEM, and AFM analyses conclusively demonstrate the successful synthesis of monodisperse HO-CDs with quasi-spherical morphology, uniform size distribution (average diameter: 3.55 ± 0.06 nm), and well-defined graphitic crystallinity. To probe the distribution of functional groups on the HO-CD surface, FTIR (Supplementary Figure S3) and XPS (Supplementary Figure S4) characterizations were conducted. These analyses reveal the presence of multiple oxygen-containing functional groups on the carbon dot surface. The existence of these functional groups not only enhances the hydrophilicity of the HO-CDs but also imparts good chemical stability and surface activity. To construct the composite catalyst, NF was introduced into the hydrothermal system as both a Ni source and substrate. This strategic design enables simultaneous petroleum coke cleavage into HO-CDs and *in situ* growth of Ni(OH)_2_ on NF within a single reaction vessel, facilitating one-step fabrication of HO-CDs-Ni(OH)_2_/NF with intimate combination.

**FIGURE 3:**
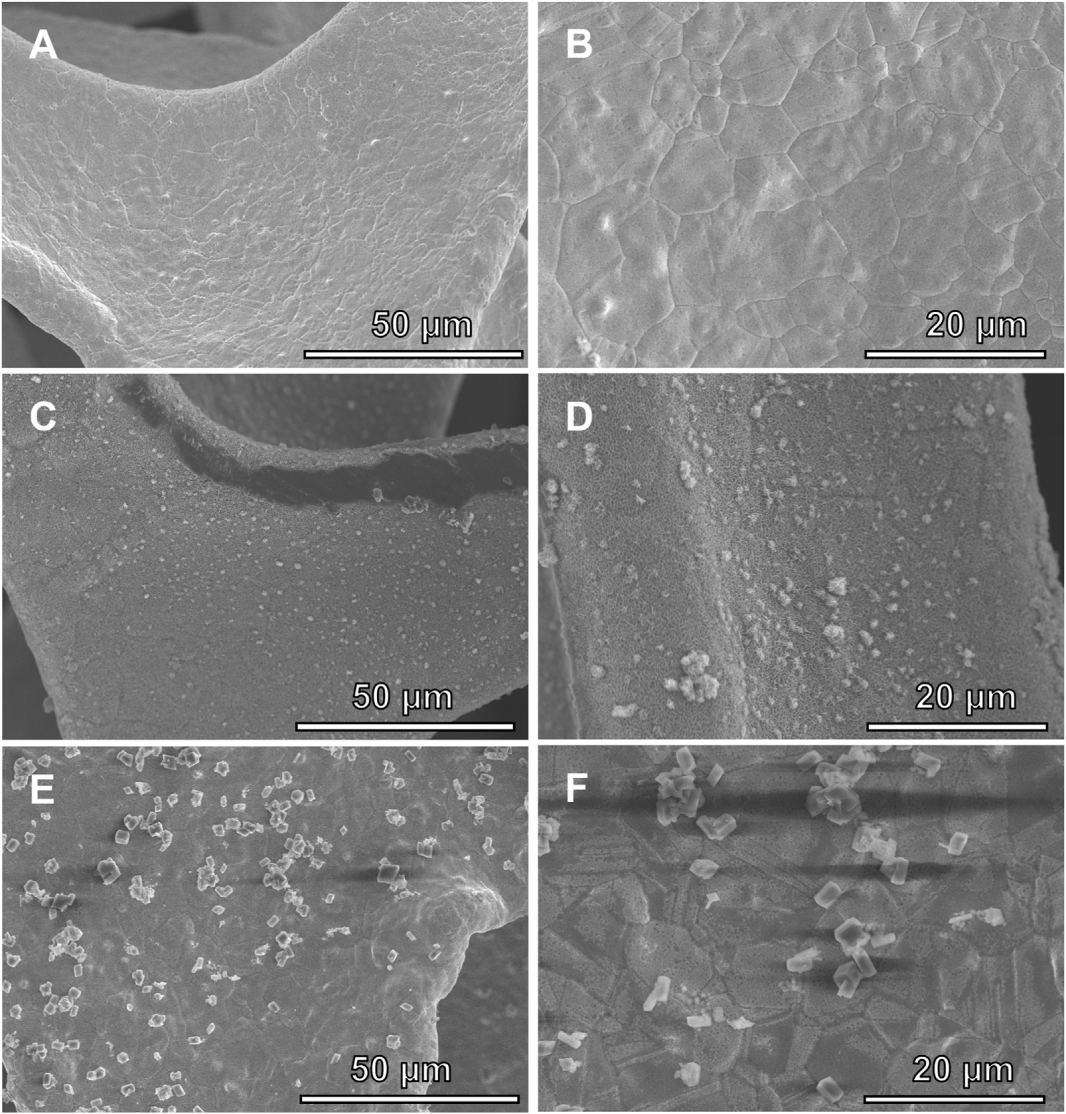
SEM images of **(A,B)** Ni(OH)_2_/NF, **(C,D)** HO-CDs + Ni(OH)_2_/NF, and **(E,F)** HO-CDs-Ni(OH)_2_/NF.

The morphology of the Ni(OH)_2_/NF, HO-CDs + Ni(OH)_2_/NF, and HO-CDs-Ni(OH)_2_/NF catalysts was characterized by SEM. As shown in Figures 3A,B, after hydrothermal treatment with H_2_O_2_ solution, the Ni(OH)_2_/NF exhibits a relatively rough surface, indicating the *in situ* growth of material on the NF scaffold during the hydrothermal process. As shown in Figures 3C,D, the *ex-situ* synthesized HO-CDs + Ni(OH)_2_/NF displays irregular distribution on the NF surface with agglomeration phenomena (Wang J. et al., 2025). In contrast, the HO-CDs-Ni(OH)_2_/NF incorporating CDs (Figures 3E,F) shows the formation of granular nanostructures on the NF surface, indicating HO-CDs effectively suppress aggregation and promote nanoscale architecture beneficial for active site exposure (Li Y. et al., 2024).

**FIGURE 4:**
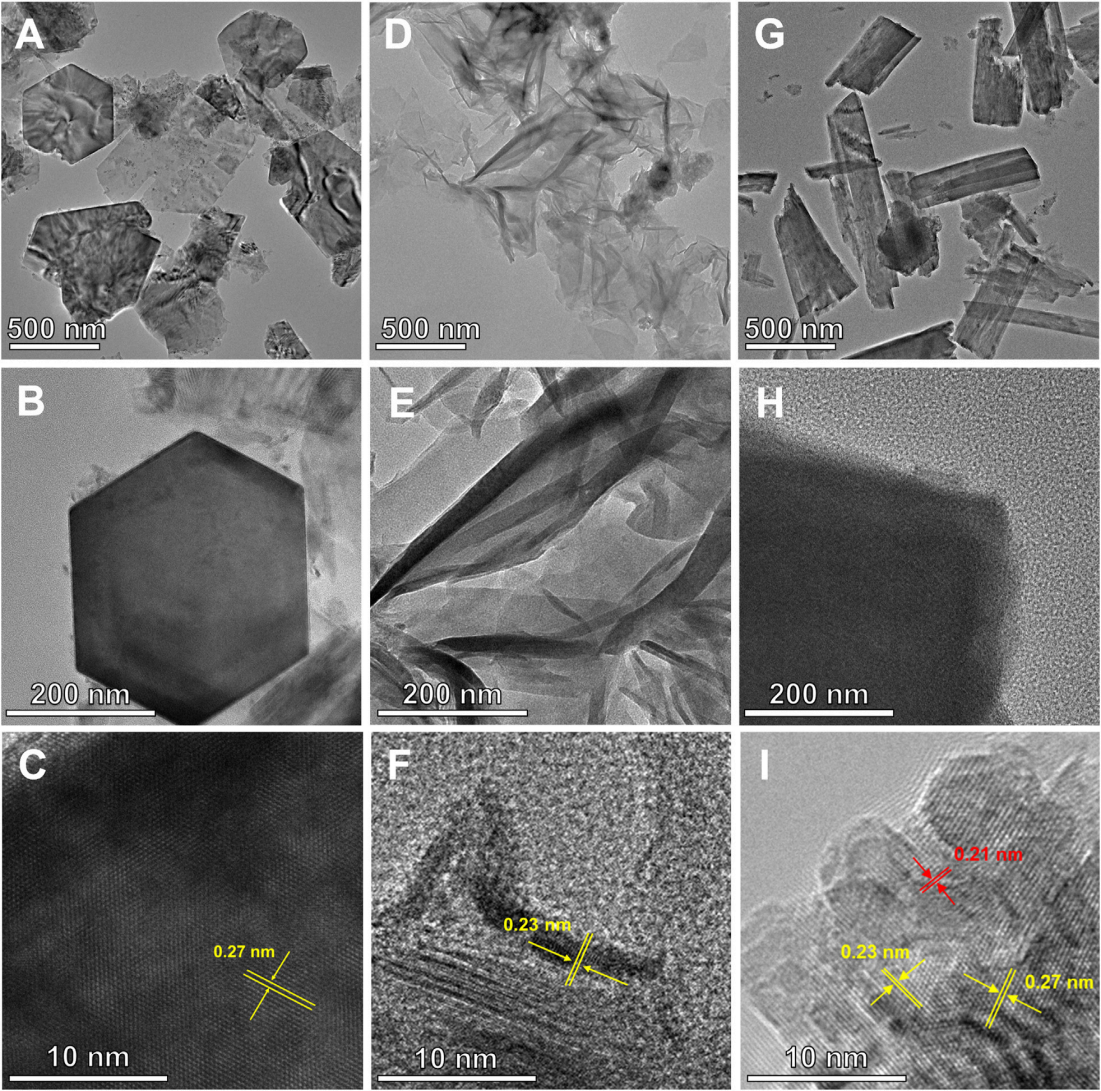
TEM images of **(A–C)** Ni(OH)_2_/NF, **(D–F)** HO-CDs + Ni(OH)_2_/NF, and **(G–I)** HO-CDs-Ni(OH)_2_/NF.

For deeper insight into the internal crystal structures of Ni(OH)_2_/NF, HO-CDs + Ni(OH)_2_/NF, and HO-CDs-Ni(OH)_2_/NF, TEM characterization was performed. As shown in Figures 4A–C, the newly formed material on the NF surface during the hydrothermal process consisted of hexagonal platelets with edge lengths ranging from 400 to 600 nm. Distinct resolvable lattice features displaying an interplanar spacing of 0.27 nm, assignable to the (100) plane of β-Ni(OH)_2_, are visible for Ni(OH)_2_/NF. Figures 4D–F reveals that HO-CDs + Ni(OH)_2_/NF exhibits an irregular morphology, with lattice fringes corresponding to an interplanar spacing of 0.23 nm, attributable to the (101) plane of β-Ni(OH)_2_. As clearly observed in Figures 4G–I, HO-CDs-Ni(OH)_2_/NF displays an irregular rectangular shape, deviating from the original hexagonal structure. This indicates that the incorporation of HO-CDs altered the growth orientation of Ni(OH)_2_ on the NF surface, suggesting that the CDs exert a modulatory effect on the material’s morphology, resulting in the formation of abundant active edges (Jiang et al., 2024). Lattice fringes with spacings of 0.23 nm and 0.27 nm, corresponding to the (101) and (100) planes of β-Ni(OH)_2_, respectively, are observed in HO-CDs-Ni(OH)_2_/NF. Additionally, distinct lattice fringes with a spacing of 0.21 nm are present, matching the (100) plane of HO-CDs, conclusively confirming the successful integration of HO-CDs with Ni(OH)_2_.

**FIGURE 5:**
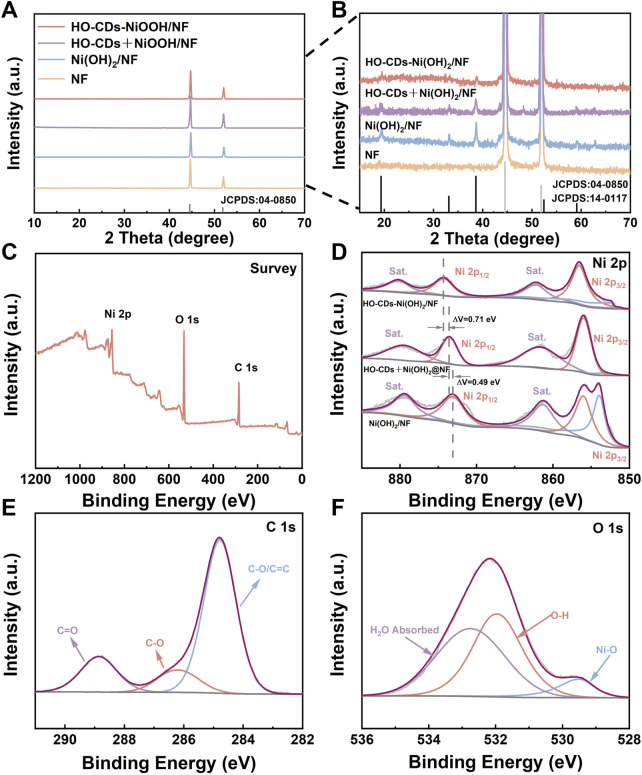
**(A,B)** XRD patterns of the various catalysts. **(C)** XPS survey spectra, **(E)** C 1s spectrum, and **(F)** O 1s spectrum of HO-CDs-Ni(OH)_2_/NF. **(D)** Ni 2p spectra of Ni(OH)_2_/NF, HO-CDs + Ni(OH)_2_/NF, and HO-CDs-Ni(OH)_2_/NF.

Bulk-phase crystal structures were elucidated through XRD analysis, with the results presented in Figures 5A,B. Apart from two distinct peaks located at 44.4° and 51.6°, corresponding to the characteristic diffraction peaks of the NF substrate (JCPDS: 04-0850), diffraction peaks appear at 2θ values of 19.6°, 33.4°, 38.8°, 52.2°, 59.2°, and 62.7° upon magnification. These peaks match the standard pattern for the hexagonal phase β-Ni(OH)_2_ (JCPDS: 14-0117), correspond to the (001), (100), (101), (012), (110), and (111) crystallographic planes, respectively. This result confirms that the hexagonal platelets grown during the hydrothermal process are the hexagonal phase β-Ni(OH)_2_. Remarkably, the XRD patterns of Ni(OH)_2_/NF and HO-CDs + Ni(OH)_2_/NF exhibit sharp peaks, indicating their high crystallinity. In contrast, HO-CDs-Ni(OH)_2_/NF shows decreased diffraction peak intensities, suggesting that the *in situ* synthesized HO-CDs exert an inhibitory effect on the crystal growth of Ni(OH)_2_.

XPS characterization resolved the elemental composition and chemical states of HO-CDs-Ni(OH)_2_/NF, with corresponding spectral data presented in Figures 5C–F. HO-CDs-Ni(OH)_2_/NF primarily comprises three elements of C, O, and Ni. In the high-resolution Ni 2p spectrum of HO-CDs-Ni(OH)_2_/NF, the two peaks located at approximately 874.1 eV and 856.8 eV correspond to Ni^2+^ 2p_1/2_ and Ni^2+^ 2p_3/2_, respectively. Satellite peaks at 880.1 eV (2p_1/2_) and 862.1 eV (2p_3/2_) are also detected. The peak appearing near 853.0 eV is attributed to Ni atoms in the NF substrate. This peak is significantly diminished in HO-CDs-Ni(OH)_2_/NF, indicating a more complete reaction on the NF surface. Compared to Ni(OH)_2_/NF and HO-CDs + Ni(OH)_2_/NF, the Ni^2+^ 2p_1/2_ peak in HO-CDs-Ni(OH)_2_/NF exhibits a positive binding energy shift of 1.2 eV and 0.71 eV, respectively. This suggests a strong electronic interaction between HO-CDs and Ni(OH)_2_, leading to electron redistribution and partial transformation of Ni species into a higher oxidation state. This electron redistribution can optimize the reaction kinetics, endowing HO-CDs-Ni(OH)_2_/NF with enhanced catalytic activity (Li T. et al., 2024). Notably, the distinct peak at 853.0 eV (Ni^0^) observed solely in Ni(OH)_2_/NF (Figure 5D) arises from the exposed NF substrate, owing to its incomplete coverage characterized by microcracks and fissures (Figures 3A,B). In contrast, the dense and uniform overlayer formed on NF in HO-CDs-Ni(OH)_2_/NF (Figures 3E,F), modulated by HO-CDs, effectively shields the underlying NF, preventing the detection of its Ni^0^ signal by XPS. In the O 1s spectrum, the two characteristic peaks near 529.4 eV and 532.8 eV can likely be attributed to Ni(OH)_2_ and physical oxygen adsorption. In the C 1s spectrum, the main forms of carbon originate from functional groups on the CDs surface. This electronic redistribution and interfacial bonding synergistically optimize adsorption energetics for enhanced OER kinetics. These collective XPS signatures consistently evidence electron transfer from Ni(OH)_2_ to oxygen-functionalized HO-CDs, which optimizes the local electronic structure for OER intermediates adsorption.

To reveal the effects of key synthetic parameters (petroleum coke dosage, hydrothermal duration, and temperature) on the electrocatalytic activity of the HO-CDs-Ni(OH)_2/_NF catalyst (Supplementary Figures S5, S6), OER measurements were conducted. The results demonstrate that the petroleum coke mass significantly affects the catalytic performance, with an optimal mass of 0.5 g. At 0.25 g, a marginal performance improvement is observed due to insufficient HO-CDs induction effect, while at 0.75 g, performance declines due to reduced oxidation degree of the NF substrate caused by excessive petroleum coke. The catalyst exhibits optimal OER performance under optimized hydrothermal conditions (140 °C, 12 h). Insufficient or excessive hydrothermal time/temperature compromises the H_2_O_2_ to ·OH conversion efficiency, suppressing the oxidative cutting of petroleum coke to form HO-CDs, thereby adversely affecting catalyst formation and performance. Catalysts prepared under the optimized conditions (0.5 g petroleum coke, 140 °C, 12 h) exhibit the most favorable structural characteristics, including the effects of CDs-modulated crystal growth and strong interfacial coupling between HO-CDs and Ni(OH)_2_ as confirmed by XRD, TEM, and XPS analyses. This synergistic optimization collectively contributes to their outstanding OER activity, achieving an overpotential of 353 mV at 50 mA cm^-2^ and a Tafel slope of 81.2 mV dec^−1^.

To investigate the effect of HO-CDs induction regulation on the electrocatalytic activity of Ni(OH)_2_, OER performance tests were conducted on the catalysts. Figure 7A presents the OER polarization curves for HO-CDs-Ni(OH)_2_/NF, HO-CDs + Ni(OH)_2_/NF, Ni(OH)_2_/NF, and NF. At a current density of 50 mA·cm^-2^, the corresponding overpotentials for HO-CDs-Ni(OH)_2_/NF, HO-CDs + Ni(OH)_2_/NF, Ni(OH)_2_/NF, and NF are 353 mV, 386 mV, 439 mV, and 518 mV, respectively. Notably, HO-CDs-Ni(OH)_2_/NF exhibits significantly enhanced performance compared to HO-CDs + Ni(OH)_2_/NF and Ni(OH)_2_/NF, with the overpotential at 50 mA·cm^-2^ reduced by 33 mV and 86 mV, respectively. To objectively evaluate the performance advantage of the HO-CDs-Ni(OH)_2_/NF catalyst, a comparative analysis of its overpotential at 50 mA cm^-2^ against representative non-precious metal OER catalysts reported in recent literature was compiled. This summary is presented in Supplementary Table S1. As shown, the HO-CDs-Ni(OH)_2_/NF catalyst exhibits promising electrocatalytic performance, demonstrating an overpotential comparable to those of recently reported efficient catalysts.

**FIGURE 6:**
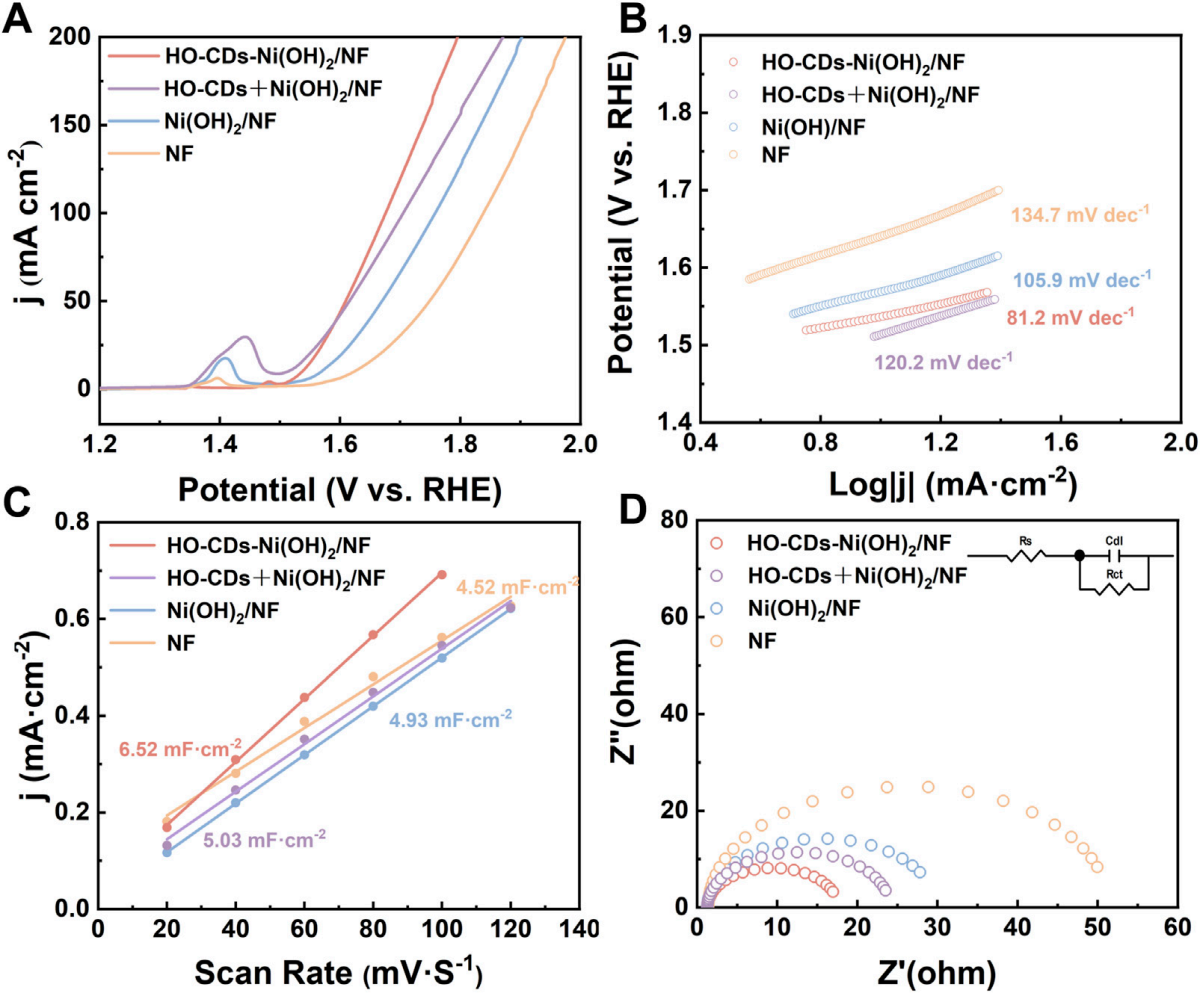
**(A)** OER polarization curves, **(B)** Tafel plots, **(C)** The C_dl_ values and **(D)** EIS Nyquist plots of NF, Ni(OH)_2_/NF, HO-CDs + Ni(OH)_2_/NF and HO-CDs-Ni(OH)_2_/NF.

This demonstrates the crucial role of HO-CDs in enhancing the performance of Ni(OH)_2_ and highlights the advantage of *in situ* HO-CDs induction on NF over the *ex-situ* induction approach. The Tafel plot (Figure 6B) is generated from the LSV data of the material. The Tafel slopes for HO-CDs-Ni(OH)_2_/NF, HO-CDs + Ni(OH)_2_/NF, Ni(OH)_2_/NF, and NF are determined to be 81.2 mV dec^−1^, 120.2 mV dec^−1^, 105.9 mV dec^−1^, and 134.7 mV dec^−1^, respectively. Among them, HO-CDs-Ni(OH)_2_/NF exhibits the lowest Tafel slope, indicating enhanced OER reaction kinetics. On the one hand, this is attributed to the particulate morphology presented on the HO-CDs-Ni(OH)_2_/NF surface, which provides more active sites for the catalytic reaction, favoring the improvement of electrocatalytic performance. On the other hand, the successful incorporation of HO-CDs enhances the material’s electrical conductivity and accelerates the charge transfer rate.

Supplementary Figures S7A-SD show the CV curves for NF, Ni(OH)_2_/NF, HO-CDs + Ni(OH)_2_/NF, and HO-CDs-Ni(OH)_2_/NF, respectively, scanned at different scan rates (20, 40, 60, 80, 100, 120 mV·s^-1^) within the non-Faradaic potential range (1.02–1.22 V vs RHE). The current density difference at 1.12 V was calculated for each sample across these scan rates. Plotting half of this current density difference against the scan rate yields the linear relationships shown in Figure 6C. The slope of the fitted line corresponds to the C_dl_ value. The electrochemical active surface area (ECSA) was calculated using the relationship ECSA = C_dl_/Cs, where Cs is the specific capacitance of a flat standard (0.022 mF cm^-2^ in 1 M KOH). The calculated ECSA values for HO-CDs-Ni(OH)_2_/NF, HO-CDs + Ni(OH)_2_/NF, Ni(OH)_2_/NF, and NF are 296.36 cm^2^, 228.64 cm^2^, 224.09 cm^2^, and 205.45 cm^2^, respectively. The largest ECSA value of HO-CDs-Ni(OH)_2_/NF confirms that the integration of HO-CDs with Ni(OH)_2_ facilitates greater exposure of active sites, significantly enhancing the electrochemical performance (Li Z. et al., 2024). The intrinsic activity of catalysts was further compared through ECSA-normalized LSV analysis. As shown in Supplementary Figure S8, HO-CDs-Ni(OH)_2_/NF exhibits slightly higher overpotential at low current densities (below 0.75 mA cm^-2^_ECSA_) due to initial activation effects, but shows substantially lower overpotential (above 0.75 mA cm^-2^_ECSA_) compared to control samples. This clearly confirms its superior intrinsic activity. The ECSA-normalized results demonstrate that the *in situ* incorporation of HO-CDs effectively generates more efficient active sites, rather than merely increasing surface area. To verify the effect of HO-CDs on the material’s electrical conductivity, electrochemical impedance spectroscopy (EIS) was performed on the samples, as presented in Figure 6D. Compared to Ni(OH)_2_/NF, HO-CDs-Ni(OH)_2_/NF exhibits a significantly lower electrochemical impedance, demonstrating that the composite formation of HO-CDs with Ni(OH)_2_ effectively enhances the charge transfer capability. This significantly reduced charge transfer resistance originates from the conductive network established by HO-CDs and their strong electronic interaction with Ni(OH)_2_, synergistically facilitating interfacial electron transfer during OER. Consequently, the material achieves higher electronic conductivity.

**FIGURE 7:**
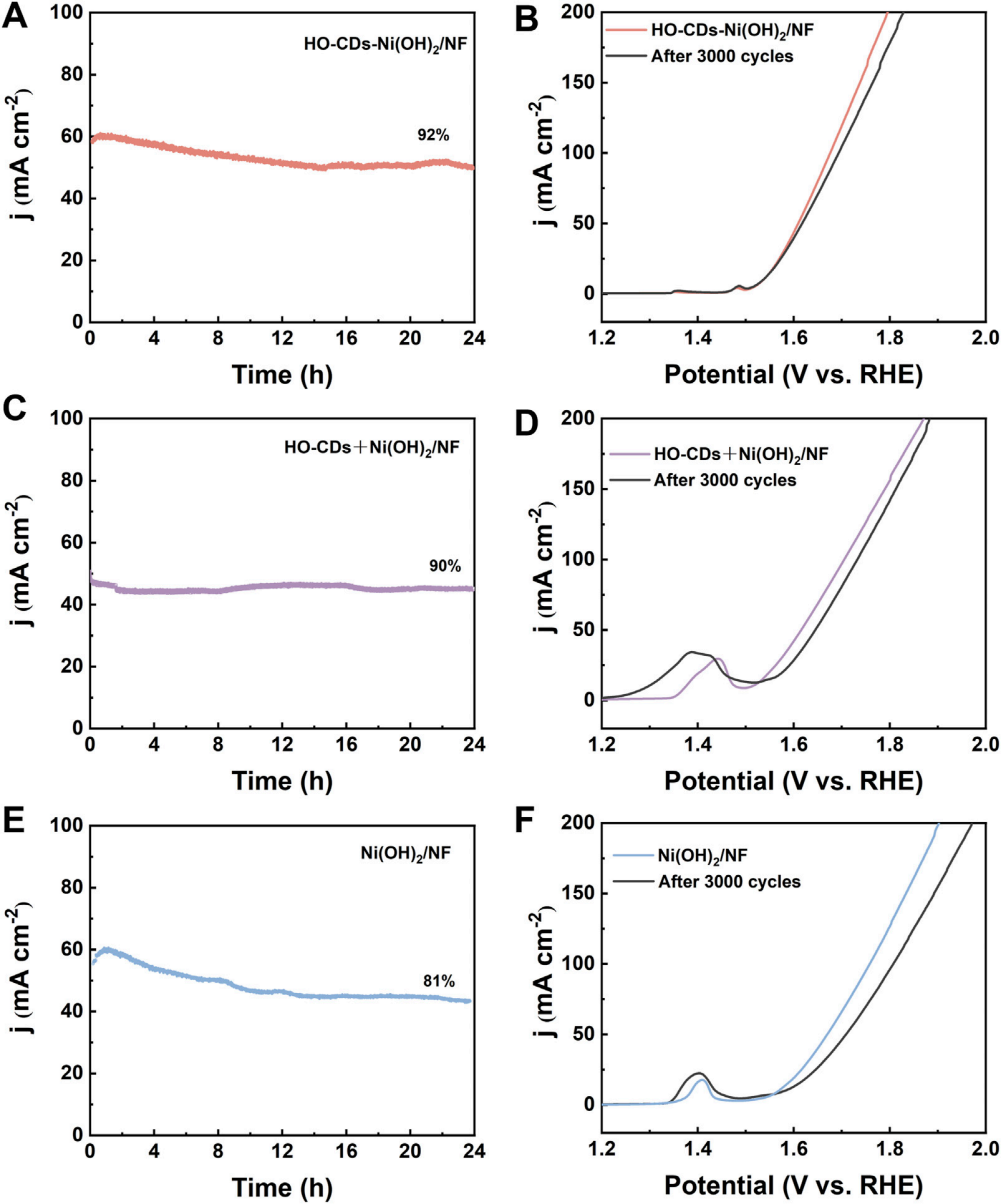
**(A,C,E)** Long-time stability test at a constant voltage for 24 h; **(B,D,F)** LSV curves before and after 3000 CV scans.

Further stability tests were conducted on the four catalysts of HO-CDs-Ni(OH)_2/_NF, HO-CDs + Ni(OH)_2_/NF, Ni(OH)_2_/NF, and NF. Chronoamperometry and CV tests were performed on each material. Figures 7A,C,E depict the variation in current density with time under constant voltage for HO-CDs-Ni(OH)_2_/NF, HO-CDs + Ni(OH)_2_/NF, and Ni(OH)_2_/NF, respectively. After a 24-h test, the current rate of HO-CDs-Ni(OH)_2_/NF reaches a high retention of 92%, while that of HO-CDS + Ni(OH)_2_/NF and Ni(OH)_2_/NF are 90% and 81%, respectively, with NF showing a retention rate of 80% (Supplementary Figure S9). This indicates that HO-CDs-Ni(OH)_2_/NF exhibits minimal decrease in current density after prolonged durability testing, demonstrating excellent long-term stability. The cyclic CV test further validates this result. Figures 7B,D,F show the LSV curves of HO-CDs-Ni(OH)_2_/NF, HO-CDs + Ni(OH)_2_/NF, and Ni(OH)_2_/NF before and after 3000 CV cycles, respectively. The overpotential increments corresponding to a current density of 100 mA·cm^-2^ were 6 mV, 24 mV, and 41 mV, respectively. HO-CDs-Ni(OH)_2_/NF shows the smallest change in overpotential before and after the test, suggests that the *in situ* introduction of HO-CDs plays a significant role in enhancing the stability of the material.

## 4 Conclusion

In summary, we demonstrate a facile one-pot hydrothermal oxidation strategy for the *in situ* construction of carbon dots/nickel hydroxide composites (HO-CDs-Ni(OH)_2_/NF) using petroleum coke as a sustainable carbon source and NF as both substrate and nickel precursor in a H_2_O_2_-mediated reaction system. The key innovation lies in utilizing in situ-generated CDs to precisely control Ni(OH)_2_ crystal growth, which suppresses oriented crystallization while creating highly active nanostructures with abundant catalytic sites. This unique design not only generates exposed active sites but also establishes efficient electron transfer pathways, collectively enhancing the intrinsic OER activity. As a result, the optimized HO-CDs-Ni(OH)_2_/NF catalyst achieves an overpotential of 353 mV at 50 mA cm^-2^, a favorable Tafel slope of 81.2 mV dec^−1^, and remarkable stability with 92% current retention during 24 h stability testing. Our work provides a universal design principle for developing high-performance transition metal electrocatalysts via *in situ* carbon nanomaterial incorporation, offering new opportunities for electrochemical energy conversion technologies.

## Data Availability

The original contributions presented in the study are included in the article/Supplementary Material, further inquiries can be directed to the corresponding author.
