# Supplementary figures and images for: One-pot hydrothermal oxidation enables in situ construction of CDs/Ni(OH)2 composite for electrocatalytic oxygen evolution
Source: Front Chem. 2025 Sep 4;13:1656451. doi: 10.3389/fchem.2025.1656451 (PMC12444632; doi:10.3389/fchem.2025.1656451)

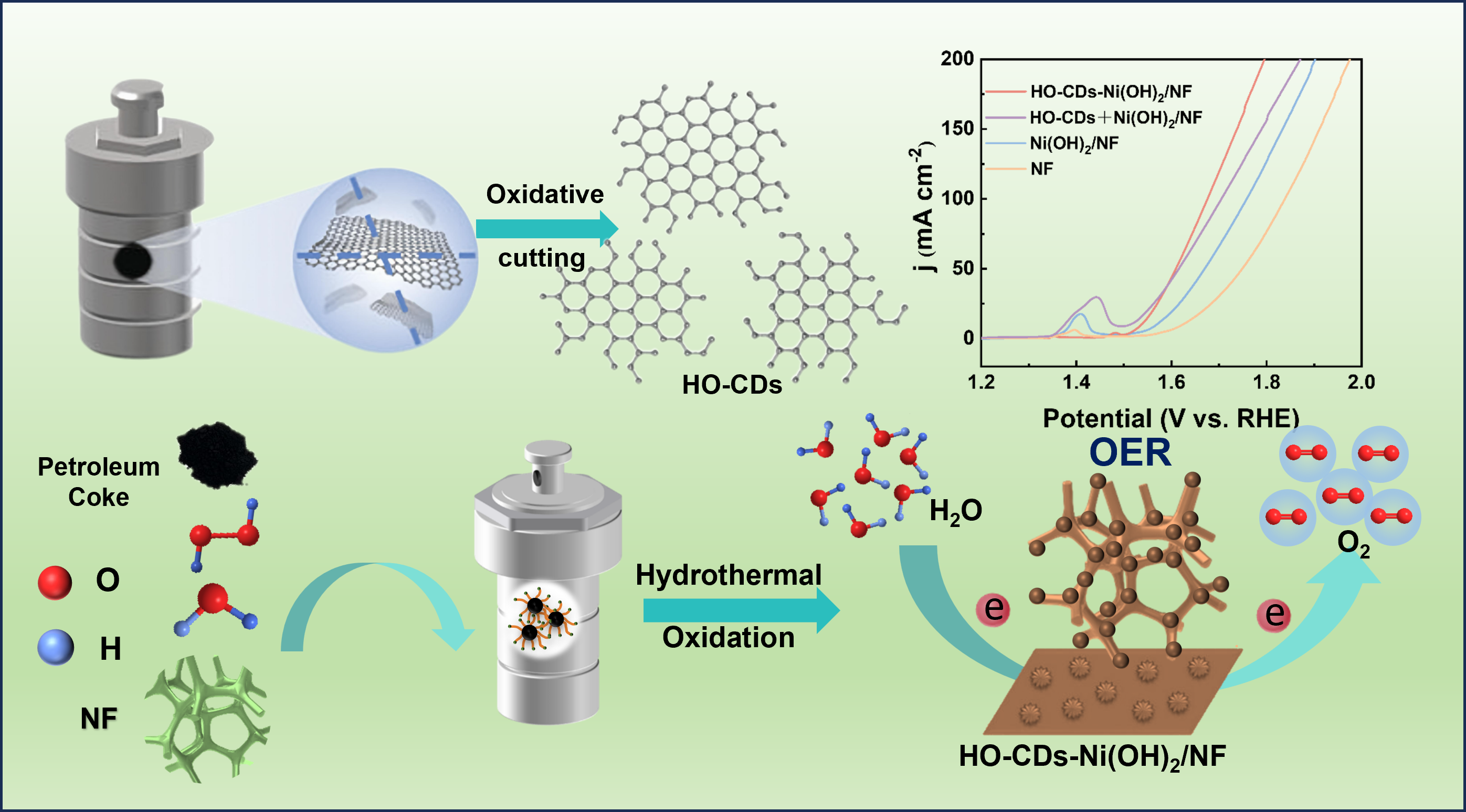

Supplement: Supplementary file 1 [file Image1.tif]
