# Supplementary material for: One-pot hydrothermal oxidation enables in situ construction of CDs/Ni(OH)2 composite for electrocatalytic oxygen evolution
Source: Front Chem. 2025 Sep 4;13:1656451. doi: 10.3389/fchem.2025.1656451 (PMC12444632; doi:10.3389/fchem.2025.1656451)
Supplement: Supplementary file 2 [file DataSheet1.docx]

Supplementary Material

**One-Pot Hydrothermal Oxidation Enables In Situ Construction of CDs/Ni(OH)_2_ Composite for Electrocatalytic Oxygen Evolution**

**Hui Wang^1^, Weijuan Xu^1^, Xuan Han^1^, Yue Yan^1^, Bingxian Zhu^1^, Zhiyuan Wang^1^, Libo Wang^1^, Qingshan Zhao^1,*^, and Mingbo Wu^1,2^**

^1^ State Key Laboratory of Heavy Oil Processing, College of Chemistry and Chemical Engineering, China University of Petroleum (East China), Qingdao 266580, China

^2^ College of Chemical Engineering, Qingdao University of Science & Technology, Qingdao 266100, China

*Corresponding author: Qingshan Zhao

E-mail address: qszhao@upc.edu.cn


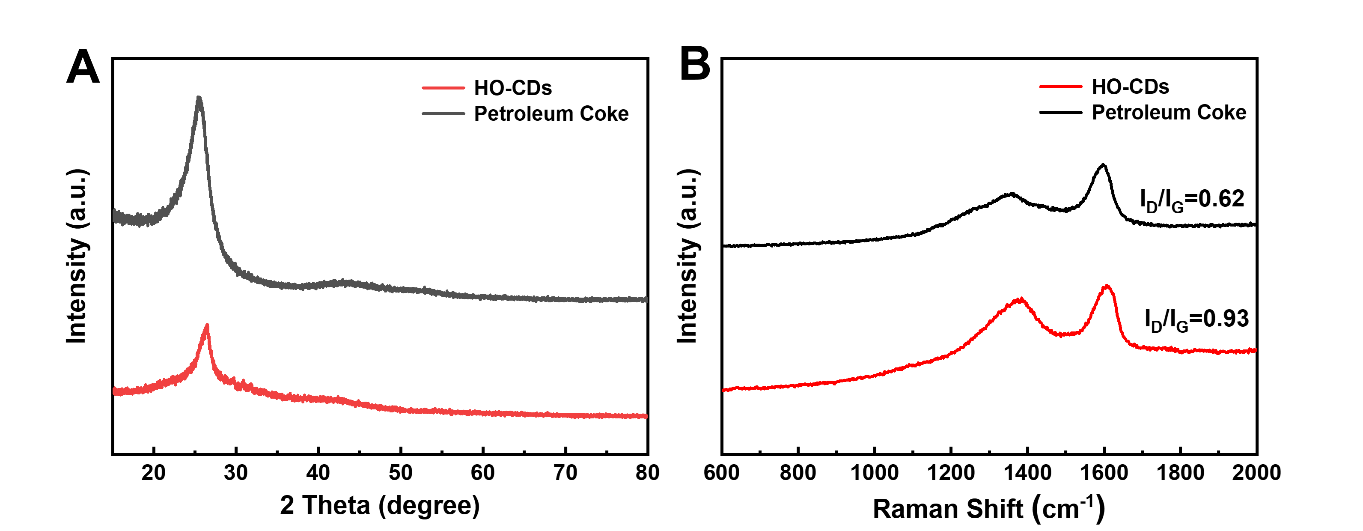


**Supplementary Figure 1** (A) XRD patterns, (B) Raman spectra of petroleum coke and HO-CDs.


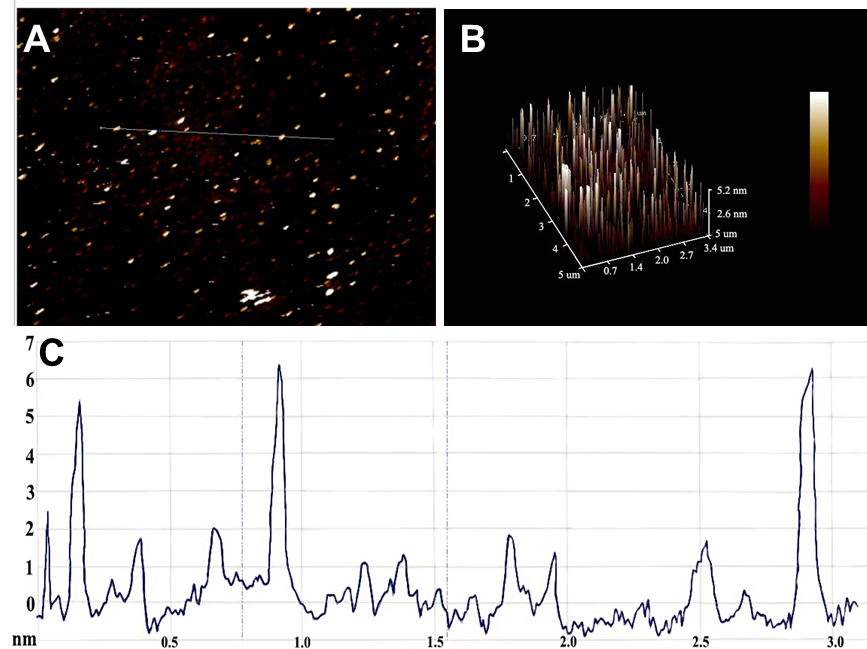


**Supplementary Figure 2** (A) 2D and (B) 3D AFM images HO-CDs. (C) The corresponding width and height profiles along the line in the 2D image of HO-CDs.


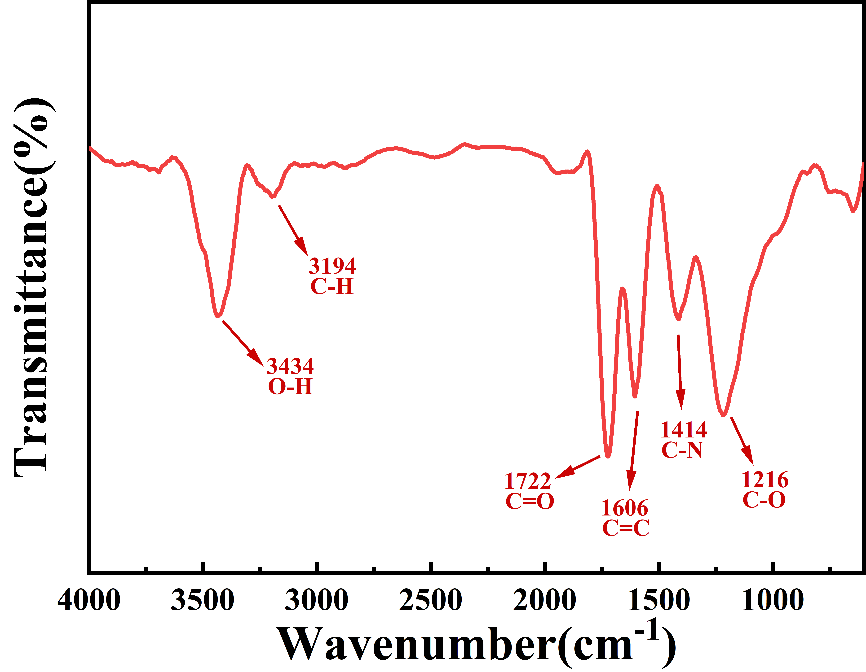


**Supplementary Figure 3** FTIR spectrum of HO-CDs.

**
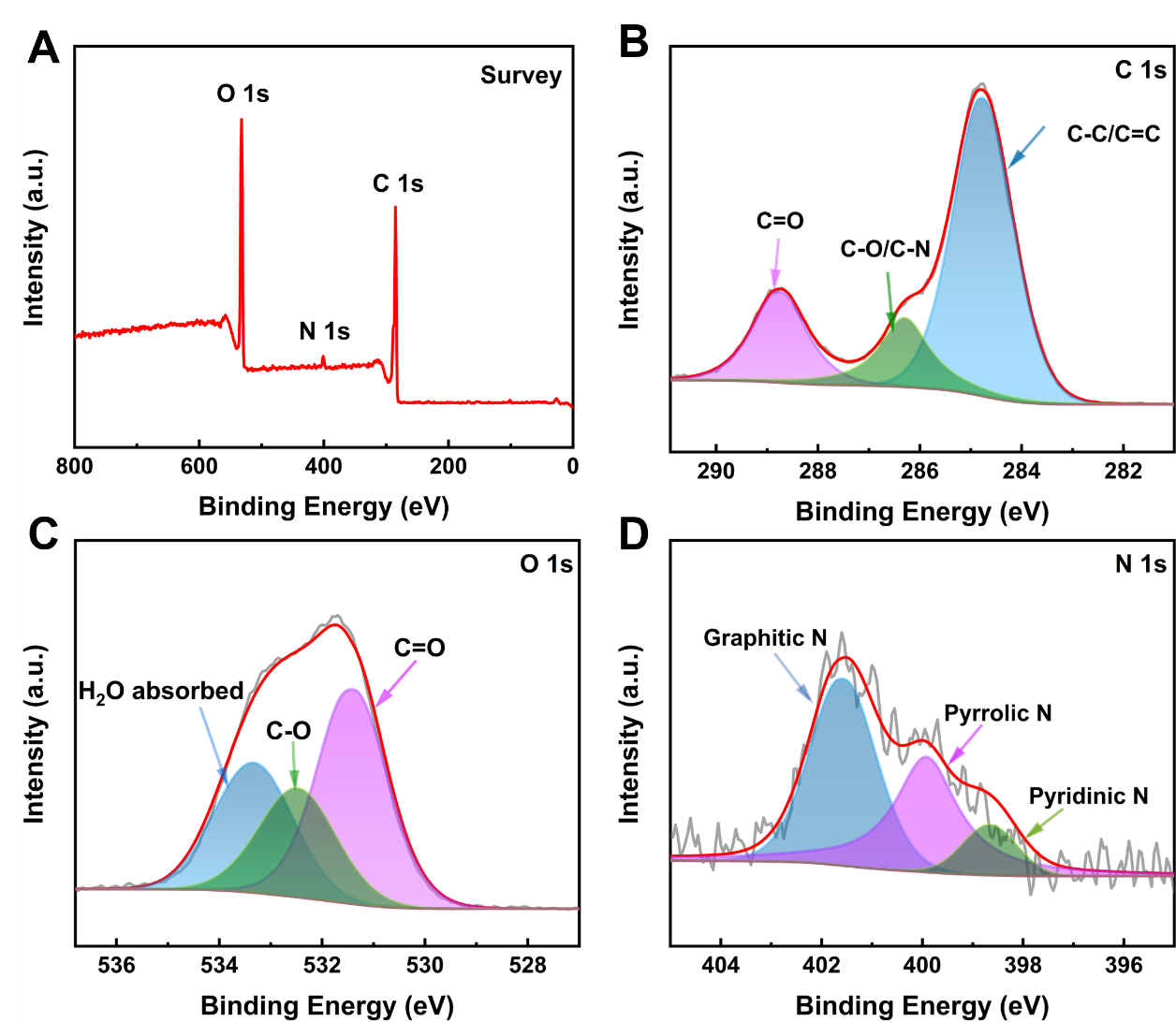
**

**Supplementary Figure 4** (A) XPS survey spectrum, (B) C 1s spectrum, (C) O 1s spectrum, and (D) N 1s spectrum of HO-CDs.

**
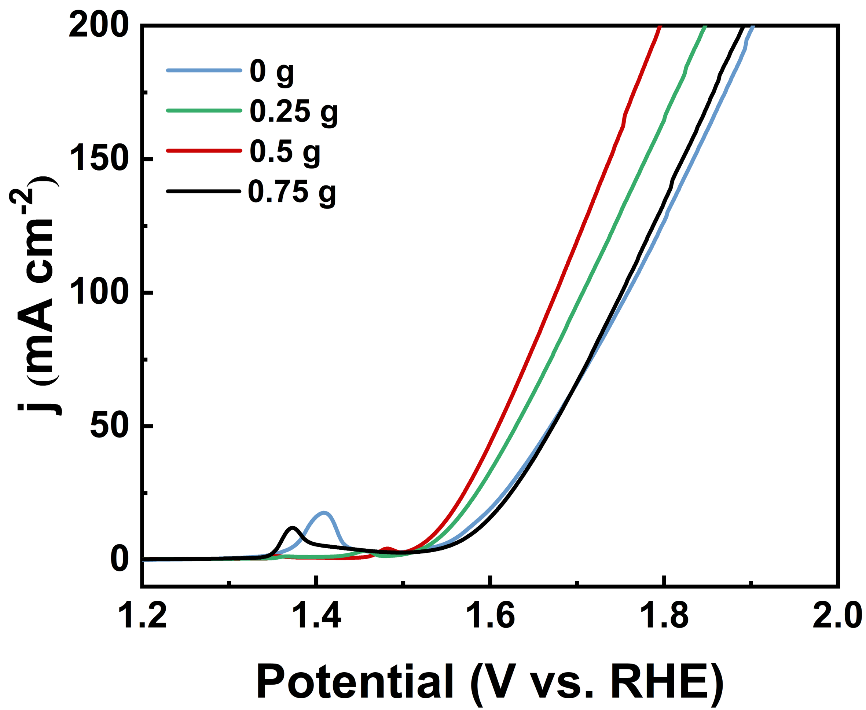
**

**Supplementary Figure 5** OER polarization curves of HO-CDs-Ni(OH)_2_/NF with different amounts of petroleum coke.


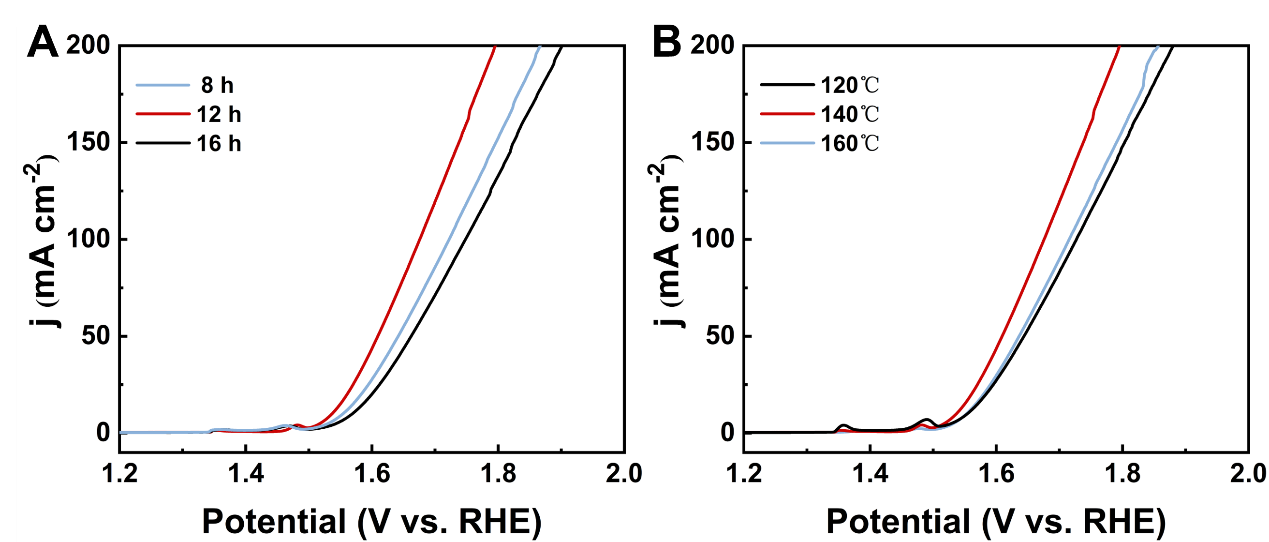


**Supplementary Figure 6** OER polarization curves of HO-CDs-Ni(OH)_2_/NF with (A) different hydrothermal times, and (B) different hydrothermal temperatures.


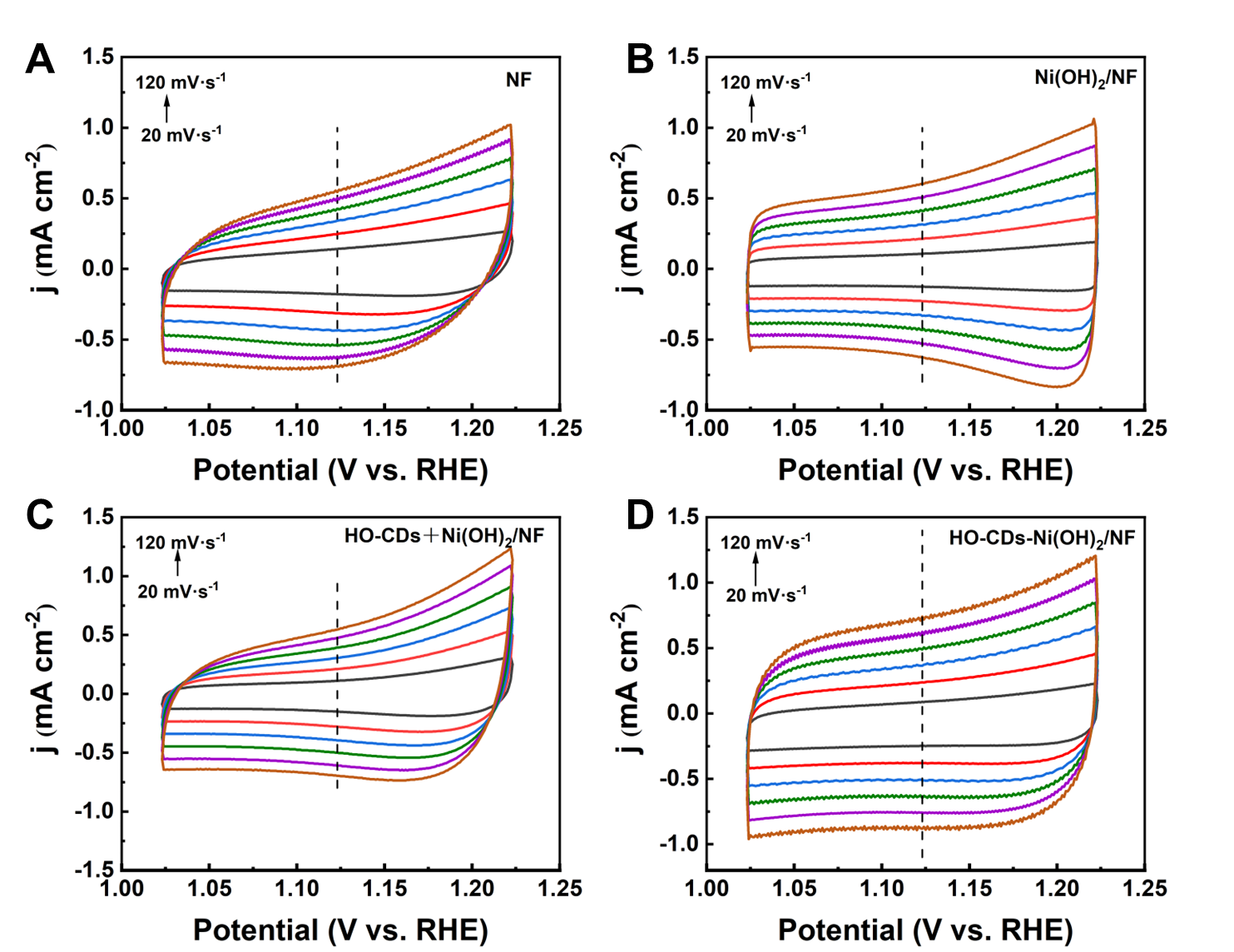


**Supplementary Figure 7** Cyclic voltammograms of (A) NF, (B) Ni(OH)_2_/NF, (C) HO-CDs+Ni(OH)_2_/NF and(D) HO-CDs-Ni(OH)_2_/NF.


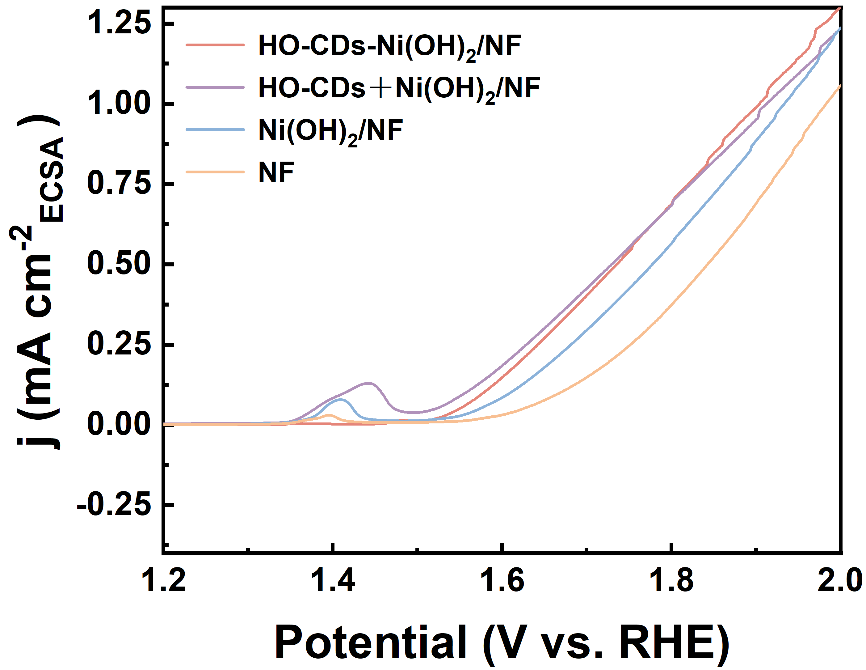


**Supplementary Figure 8**. ECSA-normalized LSV curves of NF, Ni(OH)_2_/NF, HO-CDs+Ni(OH)_2_/NF and HO-CDs-Ni(OH)_2_/NF.


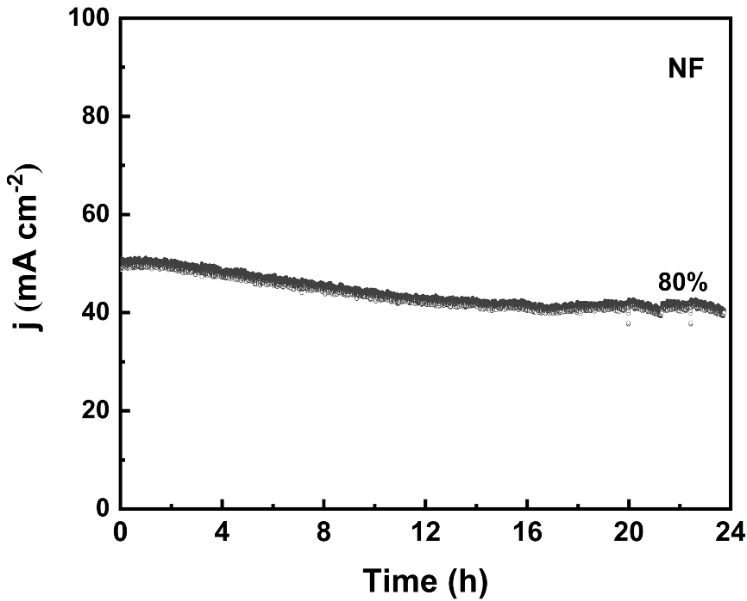


**Supplementary Figure 9** Long-term stability test at a constant voltage for 24 h of NF.

**Supplementary Table 1** OER overpotentials at 50 mA cm^-2^ for recently reported electrocatalysts.

| Catalyst | Overpotential (η) at 50 mA cm^-2^ | Ref. |
| --- | --- | --- |
| HO-CDs-Ni(OH)_2_/NF | **353 mV** | **This work** |
| NiS | 444 mV | (Dong. et al., 2022) |
| CoTBrPPc/rGO (2.5:0.5) | 391 mV | (Itagi. et al., 2025) |
| Ni-Cu/CF | 390 mV | (Liu. et al., 2025) |
| NiFe III (1:1)-LDH | 382 mV | (Huang. et al., 2022) |
| Co/C-CW | 370 mV | (Wang. et al., 2024) |
| Fe_7_S_8_/Co_9_S_8_ | 368 mV | (Guo. et al., 2025) |
| NiFeIrO_x_@PBN | 295 mV | (Zhu. et al., 2025) |

**Reference**

Dong, Y., Zhang, G., Liu, Q., Qi, C., Jiang, X., and Gao, D. (2022). Defect chemistry of NiS for oxygen evolution reaction. *Journal of Alloys and Compounds*, 923, 166438.doi: 10.1016/j.jallcom.2022.166438

Itagi, M., Imadadulla, M., Palanna, M., and Ahn, Y. H. (2025). Hybrid bromo-substituted cobalt phthalocyanine/rGO: An effective electrocatalyst for oxygen evolution reactions. *International Journal of Hydrogen Energy*, 125, 108-118.doi: 10.1016/j.ijhydene.2025.04.061

Liu, Y. H., Zeng, F. Y., Kuo, Y. S., Chen, Y., and Hsu, C. L. (2025). Boosting the HER/OER bifunctional electrocatalytic activity of Ni-Cu alloy via water-containing deep eutectic solvent system. *Journal of Materials Chemistry A*. 13, 24062-24072 doi: 10.1039/d5ta01877c

Huang, F., Yao, B., Huang, Y., and Dong, Z. (2022). NiFe layered double hydroxide nanosheet arrays for efficient oxygen evolution reaction in alkaline media. *International Journal of Hydrogen Energy*, 47(51), 21725-21735.doi: 10.1016/j.ijhydene.2022.04.296

Wang, C., Zhang, Q., Li, B., Liu, Z., He, C., Yang, G., et al. (2024). Anisotropic structural carbon skeleton decorated with Co nanoparticles towards oxygen evolution reaction. *Colloids and Surfaces A: Physicochemical and Engineering Aspects*, 684, 133228. doi: 10.1016/j.colsurfa.2024.133228

Gou, J., Li, X., Fang, T., Li, C., Ma, J., Bo, L., et al. (2025). N, S, O co-doped carbon nanofiber encapsulated Fe7S8/Co9S8 three-dimensional composite as an excellent OER electrocatalyst for water splitting. *Fuel*, 399, 135610.doi: 10.1016/j.fuel.2025.135610

Zhu, Y., Liu, C., Zhang, H., Zhou, Z., Jiang, Y., Wang, T., et al. (2024). A fibrous Ir-doped NiFeOx on two-dimensional materials for high efficiency oxygen evolution reaction (OER). *Journal of Electroanalytical Chemistry,* 967, 118424.doi: 10.1016/j.jelechem.2024.118424
